# Cathelicidin-trypsin inhibitor loop conjugate represents a promising antibiotic candidate with protease stability

**DOI:** 10.1038/s41598-017-02050-2

**Published:** 2017-06-01

**Authors:** Haining Yu, Chen Wang, Lan Feng, Shasha Cai, Xuelian Liu, Xue Qiao, Nannan Shi, Hui Wang, Yipeng Wang

**Affiliations:** 1 School of Life Science and Biotechnology, Dalian University of Technology, Dalian, Liaoning 116024 China; 2 Department of Pharmaceutical Sciences, College of Pharmaceutical Sciences, Soochow University, Suzhou, Jiangsu 215123 China

## Abstract

Cathelicidins are regarded as promising antibiotics due to their capability against antibiotic-resistant bacteria without cytotoxicity. However, some concerns about the balance of cytotoxicity and antimicrobial activity, weak stability and enzymatic susceptibility sually restrict their therapeutic use. Here, we designed a series of shortened variants, Hc1~15, based on our previously characterized Hc-CATH. Hc3, the one with the best activity, after point mutation was engineered with a trypsin inhibitor loop, ORB-C, to obtain four hybrid peptides: H3TI, TIH3, H3TIF and TIH3F. All four except TIH3 were found possessing an appreciable profile of proteases inhibitory and antimicrobial characteristics without increase in cytotoxicity. Among them, TIH3F exhibited the most potent and broad-spectrum antimicrobial and anti-inflammatory activities. Fluorescence spectroscopy has demonstrated a quick induction of bacterial membrane permeability by TIH3F leading to the cell death, which also accounts for its fast anti-biofilm activity. Such mode of antimicrobial action was mainly attributed to peptides’ amphiphilic and helical structures determined by CD and homology modeling. Besides, TIH3F exhibited good tolerance to salt, serum, pH, and temperature, indicating a much better physiological stability *in vitro* than Hc3, Most importantly, in the case of resistance against proteases hydrolysis, current hybrid peptides displayed a remarkable enhancement than their original templates.

## Introduction

In the last two decades, clinical medicine is facing an alarming circumstance of the multiple drug resistance due to the antibiotics abuse^1^, which has hastened an aggressive search for new antimicrobial agents. Cathelicidins represent a novel family of gene-encoded antimicrobial peptides in vertebrates, and play key roles in host immune response to microbial infections^2^. Due to their potent antimicrobial activities and ignorant bacterial resistance, cathelicidin-derived peptides are regarded as potential alternatives to traditional antibiotics^3^. They usually possess a broad spectrum antimicrobial activity against bacteria including clinical isolated drug-resistant strains, enveloped viruses, fungi, and even parasites^4–7^.

Cathelicidins are generally characterized by a N-terminal signal peptide, a highly conserved cathelin domain followed by a C-terminal mature peptide with remarkable structural variety^8^. Despite variation in length and primary sequence, the secondary structures of mature peptides share several common features, and accordingly they are divided into 4 groups: α-helical, loop structure, extended helical, and β-sheet with 2–3 disulfide linkages^9^. Most cathelicidins display hydrophobic and cationic traits, which bestow these small peptides a unique antimicrobial mechanism different from the traditional antibiotics, that is, cathelicidins are readily attracted by and adhere to the negatively charged bacterial membranes, and form a lipophilic anchor inducing the membrane disruption and the eventual cell death within several minutes, during which the generation of drug resistance through bacterial gene mutation can hardly take place^2, 10^.

Recently, emerging evidences suggest that besides direct antimicrobial effect, cathelicidins also possess anti-inflammatory activities in the process of pathogen infections^11, 12^. For example, human LL-37 was proved to modulate immunity during bacterial infections by recruiting neutrophils, monocytes and T-cells^13, 14^, and reducing secretion of pro-inflammatory cytokines, such as IL-6, IL-8 etc^15^. In addition, it has been reported that porcine cathelicidin, PR-39, could enhance expression of cell surface heparan sulfate proteoglycans, which are of great importance for tissue repairment^16^. Given the above multiple activities and special mechanism of action, cathelicidin-derived peptides have great potential to be exploited as medical coating materials and antimicrobial agents for controlling various infections^17, 18^.

However, some concerns such as potential cytotoxicity and easy biodegradability may ultimately impede cathelicidins from clinical applications. Therefore, various means including truncation of the natural sequence, glycosylation, cyclization, fluorination, point mutation, incorporation of non-natural residues have been used to improve antimicrobial activity whilst reduce the undesirable cytotoxicity^19–24^. For example, replacement of residues next to Arg to Trp greatly enhanced the antimicrobial activity of PMAP-36^23^; substitution of Lys with Arg in Tritrpticin was found almost no hemolytic activity whereas retaining the antimicrobial activity^25, 26^. However, these methods are time consuming and costly, and still lack the systematic rationale on the molecular design principles.

Another main obstacle impeding the application of cathelicidins is the ease degradation of peptides by innate proteases or those existing in environment, leading to low bioavailability^27^. Thus, a bifunctional peptide possessing both antimicrobial and protease inhibitory activities would be an ideal template for future clinical use of cathelicidins. Trypsin and proteinase K are two members of serine proteinase family with broad spectrum. Serine proteinase inhibitors are widely distributed in animals, plants, and microorganisms, which play a key role in combating the proteinases of pests and pathogens^28^. So far, some small serine proteinase inhibitors have been discovered, such as a 14-residue SFTI-1 from sunflower seeds and a disulfidebridged hendecapeptide (CWTKSIPPKPC) loop, called ORB-C, from frog of *Odorrana grahami*, which is the smallest serine proteinase inhibitor ever found^29, 30^.

Our previous work has characterized a novel cathelicidin (Hc-CATH) showing potent bactericidal activity from the sea snake, *Hydrophis cyanocinctus*, which consists of 30 residues and mainly adopts an alpha-helical conformation^31^. Here, we truncated its α-helix segment important for the bactericidal activity^32^, and developed a series of shortened variants, termed Hc1~15. Furthermore, we hybridized the best hit, Hc3, with ORB-C, and after point mutations eventually obtained a series of desired bifunctional peptides possessing both antimicrobial and trypsin inhibitory activities. Whether the introduction of attribute of proteases resistance helps in extending the duration of peptides’ pharmacological action was next investigated. The degradation of the designed peptides was continuously monitored by HPLC over a series of incubation time spans (0, 6, 12, 24 h) to determine their proteases stability. In light of their potentials to develop into topically used anti-infective agents or peptide-based antimicrobial biomaterials, the cytotoxicities of the hybrid peptides were investigated. Meanwhile, the anti-inflammatory activities of the hybrid peptides were also determined.

## Results

### Design and optimization of the hybrid bifunctional peptides

Derivatives of Hc-CATH, Hc1~15, were synthesized with C-terminal amidation known to influence stability and activity, and their MIC values were determined (Table 1). Almost all truncated peptides showed decreased antimicrobial activities compared with Hc-CATH, and among them Hc3 performed relatively better on both antimicrobial potency and spectrum than other analogs, while variants truncated from C-terminal showed better antimicrobial activities than those developed from N-terminal. Judging by their sequences, it might be deduced that the Phe is more important than cationic Arg and Lys in contributing to the bactericidal activity of peptide, which is further approved by the observation that Hc-4, Hc-9 and Hc-15 presented much higher MIC values in contrast to their corresponding analogs with one Phe longer, Hc-3, Hc-8 and Hc-14, respectively. In addition, it is also found that once the peptide reaches a certain minimal length of 17~18 residues, its antimicrobial activity seemingly achieves the optimum, and then drastically decreases as the sequence length gets shorter (Table 1). Note that all these analogs showed neglectable hemolytic activity (Table 2), the Hc3 was selected finally as one of the parental templates for developing bifunctional peptides.

**Table 1:** Antimicrobial activities of Hc-CATH and its truncated derivatives.

Peptide	Sequence	MIC (µg/mL)						
		*E. coli*	*S. dysenteria*	*P. mirabilis*	*P. aeruginosa*	*S. aureus*	*B. subtilis*	*C. albican*
Hc-CATH	KFFKRLLKSVRRAVKKFRKKPRLIGLSTLL	2.34	0.59	4.69	18.75	4.69	75	4.69
Hc1	KFFKRLLKSVRRAVKKFRK [NH_2_]	18.75	4.69	9.38	9.38	4.69	18.75	9.38
Hc2	KFFKRLLKSVRRAVKKFR [NH_2_]	9.38	2.34	9.38	9.38	9.38	18.75	9.38
Hc3	KFFKRLLKSVRRAVKKF [NH_2_]	18.75	2.34	4.68	9.38	9.38	9.38	9.38
Hc4	KFFKRLLKSVRRAVKK [NH_2_]	75	4.69	75	150	75	—	75
Hc5	KFFKRLLKSVRRAVK [NH_2_]	37.5	4.69	18.75	75	37.5	150	37.5
Hc6	KFFKRLLKSVRRAV [NH_2_]	75	18.75	37.5	150	75	150	75
Hc7	KFFKRLLKSVRRA [NH_2_]	—	75	—	150	—	—	—
Hc8	FFKRLLKSVRRAVKKFRK [NH_2_]	18.75	4.69	18.75	18.75	—	37.5	9.38
Hc9	FKRLLKSVRRAVKKFRK [NH_2_]	150	18.75	—	150	9.38	—	150
Hc10	KRLLKSVRRAVKKFRK [NH_2_]	—	—	—	—	—	—	—
Hc11	RLLKSVRRAVKKFRK [NH_2_]	—	150	—	—	—	—	—
Hc12	LLKSVRRAVKKFRK [NH_2_]	—	—	—	—	—	—	—
Hc13	LKSVRRAVKKFRK [NH_2_]	—	—	—	—	—	—	—
Hc14	FFKRLLKSVRRAVKKF [NH_2_]	18.75	4.69	9.38	37.5	9.38	37.5	150
Hc15	FKRLLKSVRRAVKKF [NH_2_]	75	37.5	—	—	—	—	—

MIC: minimal inhibitory concentration. These concentrations represent the mean values of three independent experiments performed in duplicate. —, no detectable activity in the inhibition zone assay at peptide dose of 2 mg/mL.

**Table 2:** Hemolysis rates of the peptides.

Conc. (μg/mL)	12.5	25	50	100	200
Hc-CATH	—	—	—	—	5.25
Hc1	1.09	2.90	3.26	4.35	8.33
Hc2	1.45	0.36	1.09	2.90	3.98
Hc3	2.17	4.35	5.43	5.80	6.88
Hc4	2.90	1.81	1.09	2.54	4.71
Hc5	3.62	1.09	2.90	5.43	5.07
Hc6	1.45	2.90	1.81	0.72	3.62
Hc7	2.17	1.45	0.72	3.26	5.07
Hc8	2.17	3.62	0.36	2.17	1.45
Hc9	3.99	2.90	2.54	2.54	4.17
Hc10	2.17	2.90	3.99	1.81	2.17
Hc11	4.71	3.26	2.90	4.71	5.80
Hc12	2.17	1.45	0.11	4.35	3.99
Hc13	1.45	1.81	0.36	1.45	4.16
Hc14	3.98	2.54	1.98	1.36	3.26
Hc15	3.62	4.35	5.07	8.70	9.06
H3TI	1.80	1.55	2.28	3.50	7.39
H3TIF	1.60	1.23	1.96	2.35	5.53
TIH3	1.94	0.94	1.76	3.17	3.08
TIH3F	4.91	2.62	5.44	6.41	10

Hemolysis rate of Hc-CATH, its truncated derivatives and four current hybrid peptides, “—” represents no assay.

Hybridization is a simple and effective strategy for the design of novel multi-functional peptides because hybrid peptides could combine the advantages of different native peptides. Considering their inherent amphipathic feature and tendency to aggregate in bulk solution, these peptides show great potentials at interfaces^33^. The disulfide-bridged hendecapeptide loop (CWTKSIPPKPC), ORB-C, also called trypsin inhibitory loop (TIL) was discovered by us previously from skin secretions of *Odorrana grahami*, and found to have strong trypsin inhibitory capability with the shortest sequence reported ever^30^. Thus, four novel hybrid peptides were designed using hybridization coupled with point mutation approaches, and the sequences with their chemical/physical parameters are shown in Table 3. The theoretically calculated molecular weights for H3TI & TIH3, and H3TIF & TIH3F are 3393.25 Da, and 3557.37 Da, respectively, and the positive net charges are all +10.

**Table 3:** Sequences and physicochemical parameters of the four hybrid peptides.

Hybrid peptide	Amino acid sequence (Length)	Mw/pI	Net charge
H3TI	KFFKRLLKSVRRAVKKF***CWTKSIPPKPC*** (28)	3393.25Da/11.21	10
H3TIF	KFFKRFFKSFRRAFKKF***CWTKSIPPKPC*** (28)	3557.37Da/11.21	10
TIH3	***CWTKSIPPKPC***KFFKRLLKSVRRAVKKF[NH_2_] (28)	3393.25Da/11.21	10
TIH3F	***CWTKSIPPKPC***KFFKRFFKSFRRAFKKF[NH_2_] (28)	3557.37Da/11.21	10

The disulfidebridged hendecapeptide (CWTKSIPPKPC) proteinase inhibitor loop is indicated in italics and bold; the Phe mutations are underlined.

### Structural characterization of the hybrid bifunctional peptides

**Figure1:**
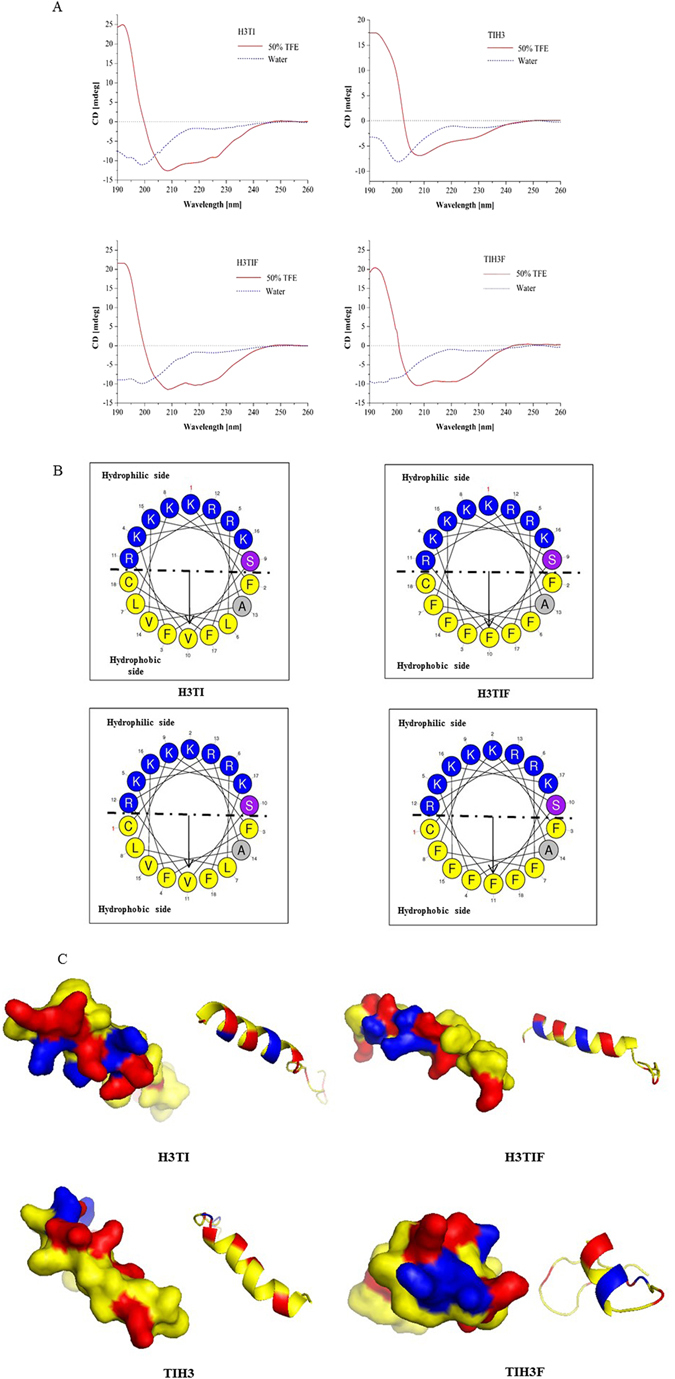
(**A**) Circular dichroism analyses of four hybrid peptides in 50% TFE solvent. (**B**) Helix-wheel plots and (**C**) tertiary structures built by homology model of four peptides. For helix-wheel plots, the hydrophobic and hydrophilic residues are separated with dash dot line, with the hydrophilic residues being concentrated on upper side of the helix and hydrophobic ones on the lower. For tertiary structures, the α-helix are displayed in the ribbon structure. Residues of Arg and Lys are displayed in blue and red, respectively in surface representatios.

The secondary structures of the hybrid peptides were investigated by CD spectroscopy. The CD spectrum of the four peptides in membrane-like environment of 50% TFE/H_2_O showed one positive band (190 nm) and two negative dichroic bands at 208 and 222 nm, consistent with the typical α -helical conformations (Fig. 1A). The helical wheel diagrams of the four hybrid peptides were subsequently plotted to estimate their amphipathicity, which revealed distinct amphipathic structures for all of them, with the hydrophobic residues facing upward, while hydrophobic ones facing downward (Fig. 1B). Such amphipathic alpha-helix structure is usually adopted by most of small cationic peptides and is thought to be important for their disrupting the membrane integrity^34^. The tertiary structures modeled via MODELLER also showed the major α-helix component for all four peptides (Fig. 1C). The positive-charged residues are distributed along the molecular surface to make the hybrid peptide electrostatic and capable to bind the negative-charged bacterial surface and LPS (Fig. 1C).

### Antimicrobial activity and bacterial killing kinetics

With ampicillin as positive control, antimicrobial activities of the hybrid peptides against Gram-negative and Gram-positive bacteria including some aquatic pathogenic bacteria, and fungi were determined using a 2-fold broth microdilution method. As summarized in Table 4, all four peptides except TIH3 displayed broad-spectrum antimicrobial activities, while TIH3 had weak effect toward only a few strains tested. Among the other three peptides, TIH3F showed the most broad-spectrum and potent antimicrobial activities, with MICs ranging from 2.37 μg/mL (toward Gram-positive *S. Aureus* 08032615 and *Nocardia asteroides* 08052412) to 37.5 μg/mL. Interestingly, H3TIF and TIH3F performed much better than Hc3 in killing seven aquatic bacteria, among which the *Vibrio Parahaemolyticus* was the most sensitive to H3TIF and TIH3F, with MICs as low as 2.37 μg/mL and 4.69 μg/mL, respectively. Results suggested that the current four hybrid peptides also are good templates in the development of novel fishery antibiotics treating infectious diseases challenging the aquaculture nowadays.

**Table 4:** Antimicrobial and trypsin-inhibitory activities of the four hybrid peptides.

Microorganisms	MIC (μg/mL)					
	H3TI	H3TIF	TIH3	TIH3F	Hc3	Ampicillin
**Gram-negative bacteria**						
*Escherichia coli* ATCC25922	37.5	37.5	—	9.38	18.75	4.69
*E. coli* 08040726	9.38	4.69	150	4.69	37.5	9.38
*E. Coli* 08032813	—	—	—	75	—	—
*E. Coli* 08032823	37.5	37.5	—	9.38	9.38	—
*E. Coli* 08040726	—	—	—	75	—	—
*Shigella dysenteriae* 0804203	9.38	4.69	—	4.69	2.34	75
*K. Pneumoniae* 08040202	—	—	—	75	75	—
*K. Pneumoniae* 08031012	—	—	—	75	75	—
*Proteus mirabilis 1376*	37.5	—	75	9.38	4.68	75
*S. Maltophilia* 090223	18.75	4.69	150	9.38	9.38	—
*P. Aeruginosa* 08021015	37.5	—	—	9.38	9.38	9.38
**Gram-positive bacteria**						
*S. Aureus* 08032706	—	—	—	75	75	18.75
*S. Aureus* 08032810	—	—	—	75	75	—
*S. Aureus* 08032615	37.5	—	37.5	2.37	9.38	9.38
*Bacillus subtilis* 08042313	9.38	37.5	—	4.69	9.38	75
*Enterococcus faecium* 08052315	9.38	9.38	—	4.69	9.38	—
*Nocardia asteroides* 08052412	18.75	4.69	18.75	2.37	4.69	—
*Staphylococcus epidermidis*	—	—	—	37.5	—	—
**Fungi**						
*Candida albicans* 08030102	4.69	4.69	—	4.69	9.38	18.75
*Candida glabrata* 08A802	4.69	9.38	—	4.69	—	18.75
*Arcyria cinerea* 08030209	—		—	37.5	—	—
**Aquatic pathogenic bacteria**						
*Vibrio harveyi*	37.5	18.75	75	9.38	18.75	37.5
*Vibrio Parahaemolyticus*	4.69	2.37	37.5	4.69	4.69	18.75
*Vibrio brasiiensis*	18.75	4.69	—	4.69	—	—
*Vibrio cholerae*	75	9.38	—	75	—	—
*Aeromonas sobria*	—	18.75	—	18.75	—	—
*Aeromonas veronii*	—	9.38	—	75	9.38	—
*Edwardsiella tarda*	37.5	—	75	37.5	—	—
**Trypsin inhibition (** ***Ki*** **)**	5.5 μM	6.5 μM	7.5 μM	1.5 μM		

MIC: minimal inhibitory concentration. These concentrations represent mean values of three independent experiments performed in duplicates. —: no detectable activity in MIC assay in dose of 200 µg/mL or in trypsin inhibition assay at concentration of 160 μg/mL.

To test the bactericidal speed of the hybrid peptides, a bacterial killing kinetic assay was performed using a colony counting method. The result indicated that all of the four peptides killed bacteria in a time-dependent manner (Table 5). H3TIF and TIH3F showed a rapid killing kinetic toward *E. coli* within 30 min (versus 3 h for ampicillin) at 5xMIC, and 2 h (versus 6 h for ampicillin) at 1xMIC. More importantly, the cfu level remained at zero when the incubation time extended to 6 h, implying that the antimicrobial effect of the two peptides was bactericidal rather than bacteriostasic.

**Table 5:** Bacterial killing kinetics of four hybrid peptides.

Time	CFU × 10^3^ (*E. coli*)							
	0 min	10 min	30 min	1 h	1.5 h	2 h	3 h	6 h
H3TI × 1	63 ± 13.4	45 ± 12.7	19 ± 7.6	17 ± 3.0	15 ± 1.6	14 ± 3.6	0 ± 0.0	0 ± 0.0
H3TI × 5	58 ± 15.6	26 ± 6.4	5 ± 2.4	0 ± 0.0	0 ± 0.0	0 ± 0.0	0 ± 0.0	0 ± 0.0
H3TIF × 1	68 ± 14.9	47 ± 8.6	37 ± 9.3	21 ± 7.2	23 ± 8.5	16 ± 5.9	8 ± 3.1	0 ± 0.0
H3TIF × 5	62 ± 13.7	9 ± 3.5	0 ± 0.0	0 ± 0.0	0 ± 0.0	0 ± 0.0	0 ± 0.0	0 ± 0.0
TIH3 × 1	60 ± 13.6	54 ± 20.7	45 ± 12.3	27 ± 6.9	19 ± 2.8	15 ± 3.5	5 ± 1.6	0 ± 0.0
TIH3 × 5	57 ± 12.3	32 ± 11.3	12 ± 5.8	2 ± 0.6	0 ± 0.0	0 ± 0.0	0 ± 0.0	0 ± 0.0
TIH3F × 1	65 ± 14.9	49 ± 12.7	24 ± 6.5	17 ± 4.5	12 ± 4.8	5 ± 2.1	0 ± 0.0	0 ± 0.0
TIH3F × 5	61 ± 13.8	16 ± 6.7	3 ± 1.0	1 ± 1.0	0 ± 0.0	0 ± 0.0	0 ± 0.0	0 ± 0.0
Ampicillin × 1	70 ± 16.3	63 ± 18.4	57 ± 9.4	47 ± 8.6	37 ± 5.5	29 ± 3.7	24 ± 1.5	0 ± 0.0
Ampicillin × 5	64 ± 15.4	46 ± 19.3	54 ± 3.5	24 ± 3.6	11 ± 4.7	3 ± 1.0	1 ± 1.0	0 ± 0.0
Water	56 ± 14.6	78 ± 16.8	131 ± 23.6	178 ± 17.9	286 ± 35.7	469 ± 56.8	1358 ± 172.6	18345 ± 345.3
	**CFU × 10** ^**3**^ **(** ***S. aureus*** **)**							
H3TI × 1	60 ± 15.6	55 ± 7.4	45 ± 9.8	32 ± 6.0	23 ± 5.3	15 ± 4.3	0 ± 0.0	0 ± 0.0
H3TI × 5	59 ± 8.3	21 ± 5.2	1 ± 1.0	0 ± 0.0	0 ± 0.0	0 ± 0.0	0 ± 0.0	0 ± 0.0
TIH3 × 1	64 ± 17.1	69 ± 15.2	54 ± 9.8	36 ± 8.8	32 ± 6.1	22 ± 3.5	8 ± 2.0	0 ± 0.0
TIH3 × 5	59 ± 14.2	36 ± 13.5	8 ± 2.6	5 ± 1.7	1 ± 0.5	0 ± 0.0	0 ± 0.0	0 ± 0.0
TIH3F × 1	69 ± 13.2	57 ± 6.2	30 ± 6.4	21 ± 5.7	11 ± 2.8	3 ± 1.0	0 ± 0.0	0 ± 0.0
TIH3F × 5	63 ± 7.5	16 ± 7.7	1 ± 0.6	0 ± 0.0	0 ± 0.0	0 ± 0.0	0 ± 0.0	0 ± 0.0
Ampicillin × 1	62 ± 17.6	66 ± 15.4	63 ± 15.8	58 ± 14.8	48 ± 8.2	31 ± 3.4	23 ± 2.6	5 ± 1.0
Ampicillin × 5	58 ± 13.6	52 ± 16.2	43 ± 9.3	39 ± 14.5	13 ± 3.8	2 ± 1.0	0 ± 0.0	0 ± 0.0
Water	66 ± 15.4	82 ± 26.4	130 ± 44.2	244 ± 38.3	329 ± 29.0	608 ± 82.3	1937 ± 242.8	21863 ± 683.5
	**CFU × 10** ^**3**^ **(** ***V. parahaemolyticus*** **)**							
H3TI × 1	65 ± 10.5	49 ± 6.6	45 ± 7.5	32 ± 6.0	23 ± 4.8	12 ± 2.6	0 ± 0.0	0 ± 0.0
H3TI × 5	60 ± 9.5	15 ± 5.8	2 ± 1.0	0 ± 0.0	0 ± 0.0	0 ± 0.0	0 ± 0.0	0 ± 0.0
H3TIF × 1	66 ± 15.6	59 ± 7.5	47 ± 8.5	36 ± 7.5	19 ± 3.6	0 ± 0.0	0 ± 0.0	0 ± 0.0
H3TIF × 5	62 ± 14.6	8 ± 1.3	0 ± 0.0	0 ± 0.0	0 ± 0.0	0 ± 0.0	0 ± 0.0	0 ± 0.0
TIH3 × 1	63 ± 16.3	47 ± 14.9	38 ± 7.5	25 ± 3.6	22 ± 6.6	5 ± 2.0	2 ± 0.8	0 ± 0.0
TIH3 × 5	58 ± 13.7	32 ± 15.4	7 ± 2.3	3 ± 1.7	0 ± 0.0	0 ± 0.0	0 ± 0.0	0 ± 0.0
TIH3F × 1	63 ± 7.9	44 ± 8.5	36 ± 5.5	21 ± 3.2	13 ± 1.7	2 ± 1.0	0 ± 0.0	0 ± 0.0
TIH3F × 5	61 ± 8.3	19 ± 7.9	1 ± 0.3	0 ± 0.0	0 ± 0.0	0 ± 0.0	0 ± 0.0	0 ± 0.0
Meropenem × 1	62 ± 17.8	66 ± 18.5	57 ± 17.6	46 ± 15.2	44 ± 3.6	37 ± 4.5	33 ± 5.2	2 ± 1.0
Meropenem × 5	57 ± 14.1	42 ± 17.9	31 ± 12.5	22 ± 11.4	15 ± 3.8	2 ± 1.4	0 ± 0.0	0 ± 0.0
Water	62 ± 18.2	84 ± 25.9	128 ± 42.1	254 ± 37.9	335 ± 28.4	612 ± 72.6	1756 ± 235.4	21786 ± 674.9

CFU: colony forming unit; 1xMIC and 5xMIC of samples were used in this experiment; The MICs of H3TI, H3TIF, TIH3, TIH3F and ampicillin against *E. coli* 08040726 are 9.38, 4.69, 150, 4.69 and 0.12 µg/mL, respectively; H3TI, H3TIF, TIH3, TIH3F and meropenem MICs to *V. parahaemolyticus* are 4.69, 2.37, 37.5, 9.38 and 0.47 µg/mL, respectively.

### Effect of hybrid peptides on *C. albicans* biofilms

**Figure 2:**
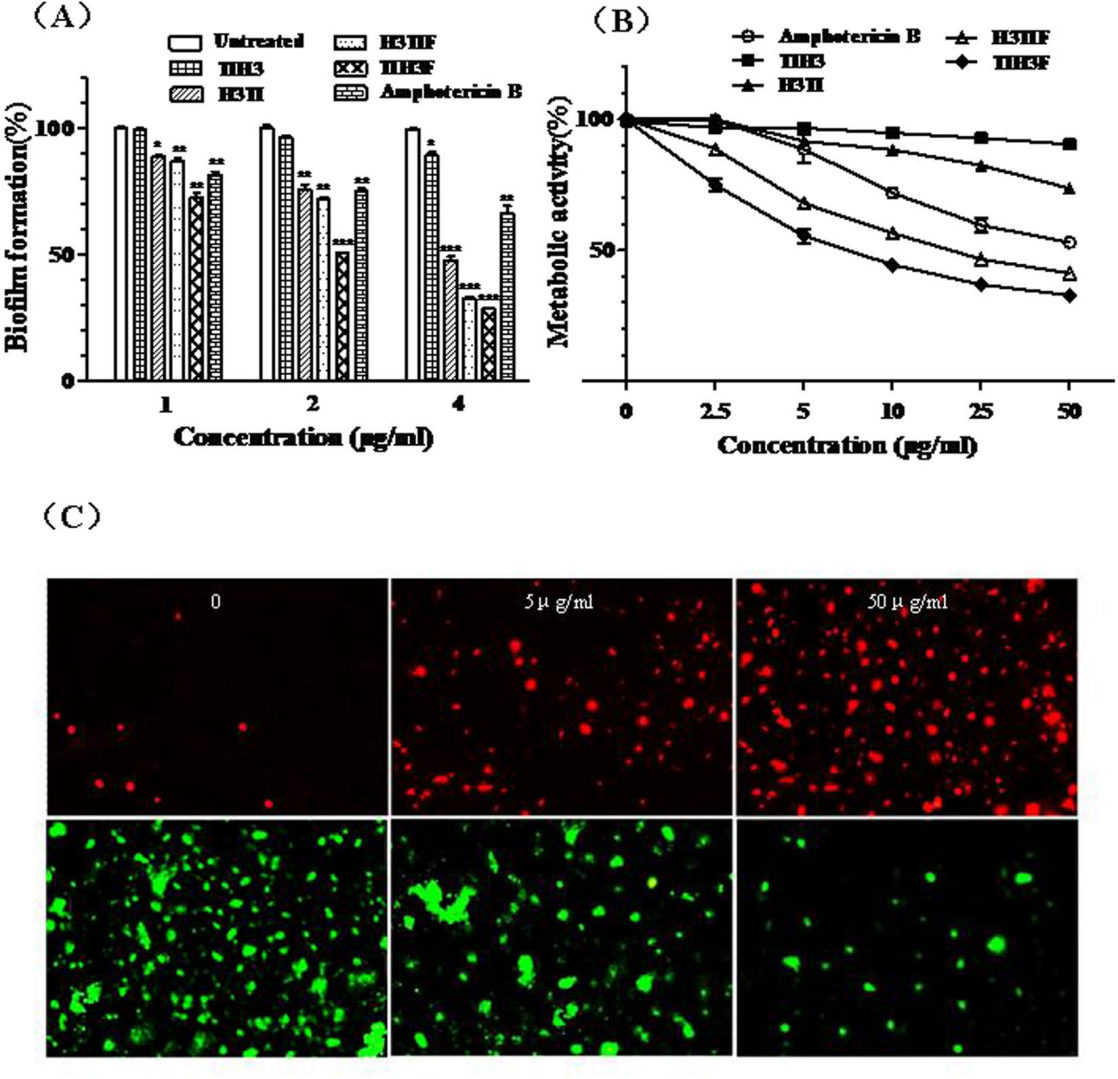
Activities of TIH3, H3TI, H3TIF and TIH3F inhibiting *C. albicans* biofilms formation and killing *C. albicans* in established biofilms. (**A**) The inhibitory effect of TIH3, H3TI, H3TIF and TIH3F against *C. albicans* biofilm formation. *C. albicans* was grown in a 6-well plate with coverslip at 35 °C for 36 h in the presence of different concentrations of four peptides. Growth in control is set to 100% and percent of biofilm formed is indicated in different bar. Data are presented as the mean ± SD from three independent experiments (*P < 0.05; **P < 0.01; ***P < 0.001; by unpaired t test). (**B**) The killing effect of four hybrid peptides against *C. albicans* in the biofilms. Cells treated with peptides and amphotericin B of series concentrations, with negative control set to a 100%. Data are presented as the mean ± SD from three independent experiments. (**C**) Visualization of TIH3F killing *C. albicans* in established biofilms photographed by fluorescence microscope. Living cells stained by SYBR Green I are green and dead cells stained by PI are red.

Biofilms are found involved in a wide variety of microbial infections, by one estimate 80% of all infections. The effects of the four peptides on the *C. Albicans* biofilm formation were examined by measuring the percentage of biofilm formation. The biofilm treated with TIH3F and H3TIF was obviously decreased than untreated and those treated with the other two peptides and amphotericin B (2 μg/mL). Moreover, this inhibitory activity was dose-dependent with the maximal inhibition of 60% observed under TIH3F (4 μg/mL) treatment, while amphotericin B at 4 μg/mL only exerted 25% inhibition (Fig. 2A), suggesting that TIH3F and H3TIF could significantly inhibit the formation of *C. albicans* biofilm at sub-MIC concentrations.

To determine the effect of TIH3F on established *C. albicans* biofilm, both TIH3F and amphotericin B of series concentrations were added, and metabolic activity was decided by XTT assay. The biofilm was damaged by 50% under treatment of 5 μg/mL TIH3F, while that incubated with 5 μg/mL amphotericin B still remained intact. H3TIF in a very low dose of 5.0 μg/mL also showed evident antifungal activity within the biofilm (Fig. 2B). The wrecking effects of TIH3F and H3TIF against the established biofilm were demonstrated as dose-dependent. In order to visually confirm this effect, the preformed biofilms treated with TIH3F of series concentrations were further stained with PI/SYBR Green I and photographed by fluorescence microscopy. The green color representing cell viability reduced significantly as TIH3F’s concentration increased, whilst the red dye that stains only dead cells with broken membranes was augmented a lot (Fig. 2C). This result is coherent with fungi metabolic assay, indicative of the significant decline of fungi metabolic activities induced by TIH3F and H3TIF. Current results demonstrated that TIH3F and H3TIF can effectively kill *C. albicans* in preformed biofilm, and finally destroy all of the cell membranes once the peptide’s concentration reaches 50 μg/mL, because the red stain nearly occupied all vision (Fig. 2C).

### Trypsin inhibition assay

At concentration of 160 μg/mL, all of the four hybrid peptides effectively inhibited the forming of macroscopic yellow product generated by tryptic hydrolysis of NBLAN. The inhibition constants (*Ki*) are of the same order, ranging from 1.5 to 5.5 μM (Table 4), suggesting that the current four hybrid peptides all possess strong trypsin inhibition activity that bestows them the attribute of enzyme stability.

### Hemolytic and cytotoxic assays

The hemolytic and cytotoxic activities of the four hybrid peptides were examined against the fresh-prepared human erythrocytes and normal cell line HUVEC (human endothelial cells), respectively. At a concentration up to 200 μg/mL (nearly 10-fold high than their MICs of most tested microorganisms), the four hybrid peptides only induced 3~10% hemolysis (Table 2) and showed neglectable cytotoxicities (data now shown).

### Stability evaluation of the hybrid peptides

**Figure 3:**
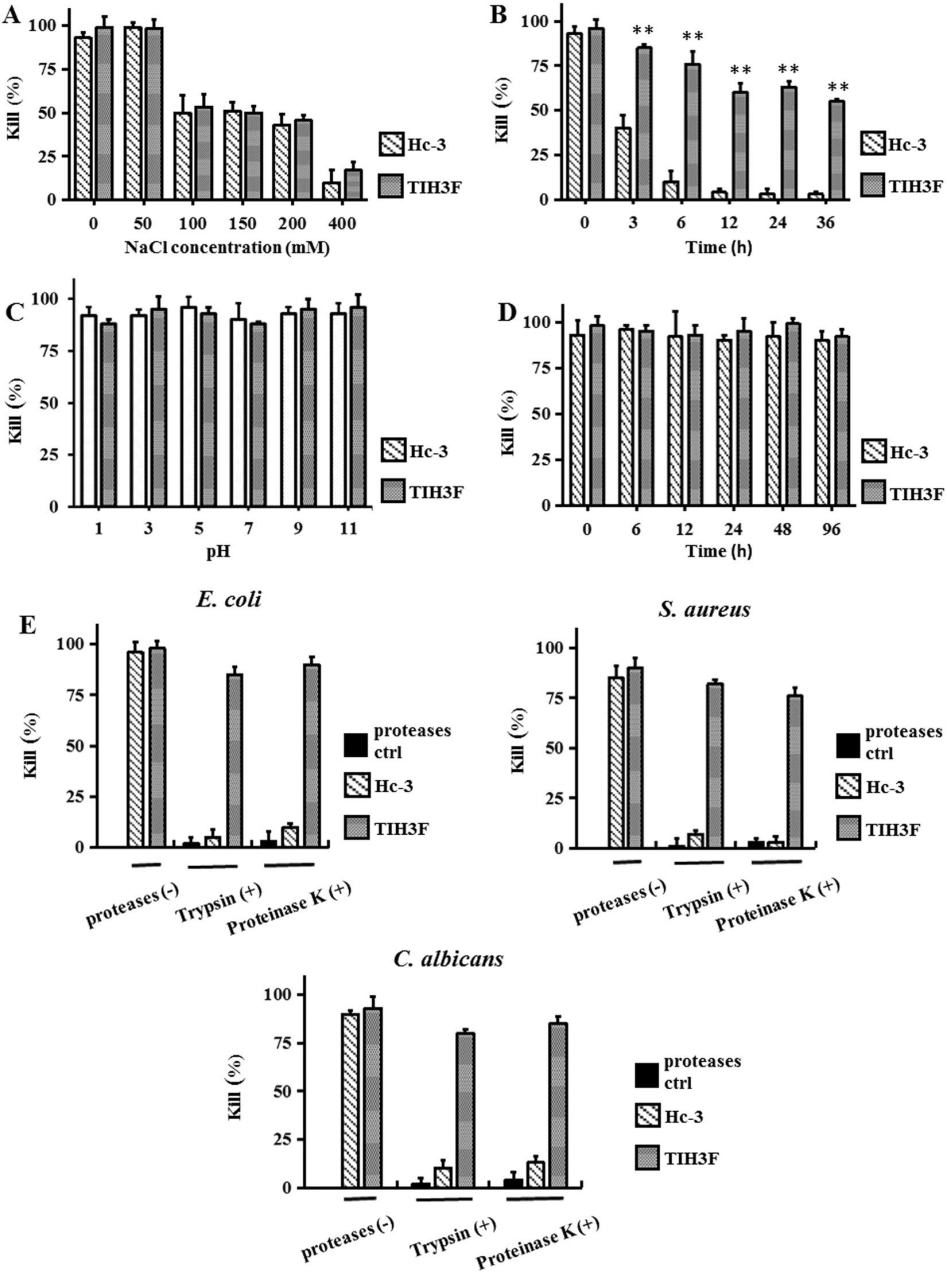
(**A**) Effects of various concentrations of NaCl on the antibacterial activity of Hc-3 and TIH3F against *E. coli*. (**B**) Stability of Hc-3 and TIH3F in human serum (20%). (**C**) Effects of pH value on the antibacterial activity Hc-3 and TIH3F against *E. coli*. (**D**) Thermal stability of Hc-3 and TIH3F at normal human body temperature of 37 °C. (**E**) Antimicrobial activities of Hc-3 and TIH3F under the influence of proteases (trypsin and proteinase K) against *E. coli*, *S. aureus* and *C. albicans*. (**F**) The degradation rates of Hc-3 and TIH3F after incubation with proteases (trypsin and proteinase K). Three separate experiments were performed to calculate the mean ± SD (*P < 0.05; **P < 0.01; by unpaired t test).

It has been reported that the antimicrobial activities of many AMPs can be significantly abolished by the presence of salt^35^. Therefore, we investigated the effect of salt on the antimicrobial activity of the TIH3F, the one with the best killing effect among the four, using Hc-3 as control. As shown in Fig. 3A, under the presence of 50 mM NaCl, the TIH3F’s antimicrobial activity was not affected as Hc-3, and the percentage of bacteria killed remained approximately the same as that in a non-salt solution. As the NaCl concentration increased from 100 mM up to 200 mM, much higher than human physiological salt concentration of 150 mM, the kill (%) declined by half, suggestive of the high salt-resistance of TIH3F.

Peptide degradation by proteases and their interaction with serum components usually affect the antimicrobial activity of AMPs. Therefore, the serum stability of TIH3F was tested in the present study. After incubating with 20% fresh human serum for up to 36 h, the kill (%) only gradually decreased to 75% (6 h),then to 65% (36 h) (Fig. 3B), whilst Hc3’s bactericidal ability dropped more than half after 3 h and almost diminished after 6 h incubation, implying the significant serum stability enhancement of TIH3F compared with its parental template Hc-3. Besides, TIH3F retained its potent antimicrobial activity even after incubation with human serum for 36 h, far more stable than the previously described cathelicidins^36^ and Hc-CATH^31^, indicating its potential for systemic therapeutic applications.

To show the effects of physiological condition on the antimicrobial activity of the peptides, we also examined the killing effects of TIH3F and Hc-3 against *E. coli* under pH values ranging from 1.0–11.0. The result demonstrated that the antimicrobial activities of both peptides were unaffected under such violent pH fluctuation (Fig. 3C), indicating the significant pH-tolerance of TIH3F and Hc-3, even under the extreme pH values of 1 and 11.

In addition to the above chemical and biological factors, as an important class of natural bioactive molecules that are composed of amino acids, AMPs are also susceptible to many physical factors, such as thermal factor. As shown in Fig. 3D, after incubation in human physiological temperature (37 °C) for up to 96 h, TIH3F maintained its potent antimicrobial activity as its parental template, Hc3.

**Figure 4:**
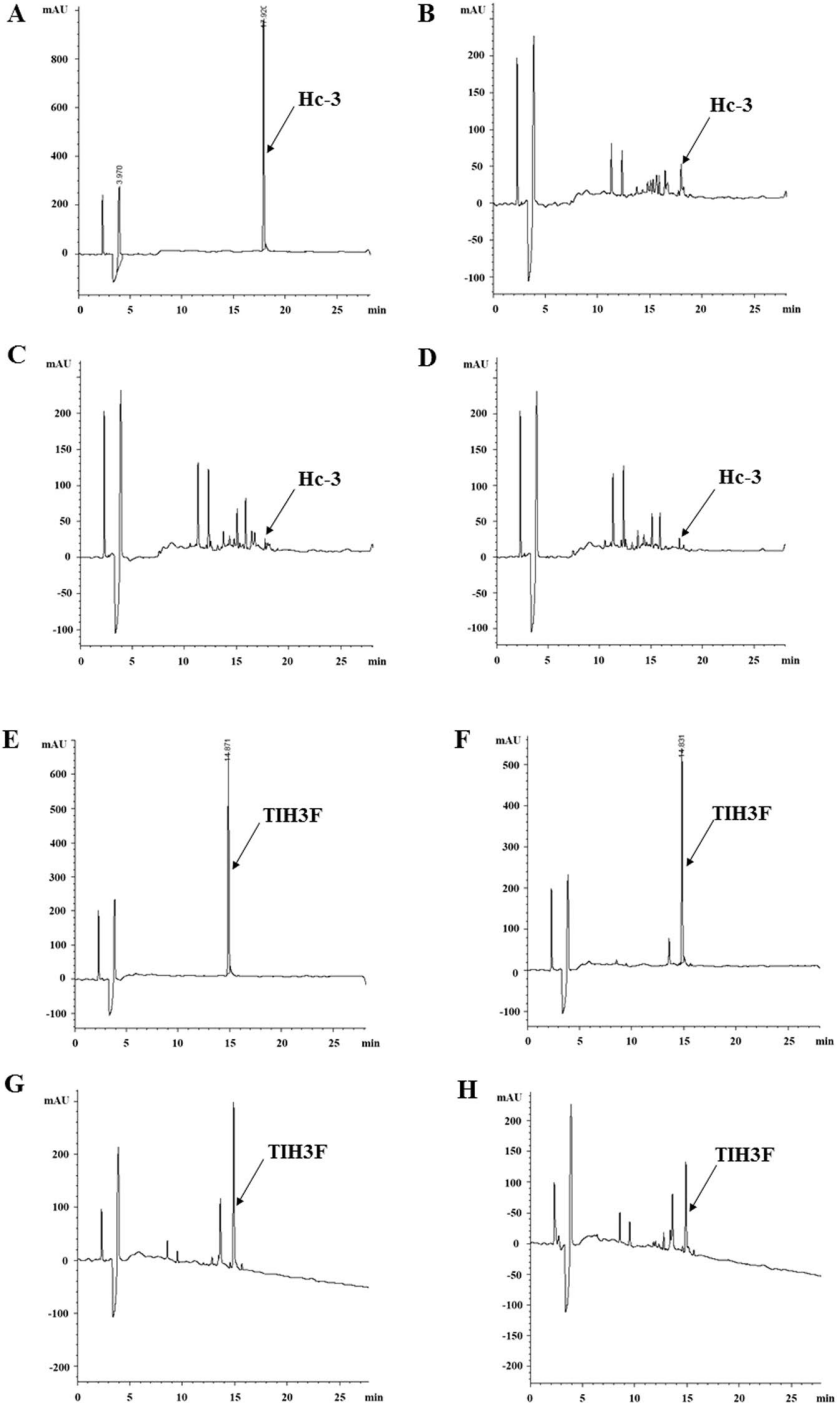
Analytical reversed-phase HPLC traces of Hc-3 and TIH3F after treatment with trypsin for different times. (**A**) LC analysis of pure Hc-3. LC analysis of Hc-3 after incubation with trypsin at 37 °C for (**B**) 6 h (**C**) 12 h and (**D**) 24 h. (**E)** LC analysis of pure TIH3F. LC analysis of TIH3F after incubation with trypsin at 37 °C for (**F**) 6 h (**G)**12 h and (**H**) 24 h.

To evaluate the resistance of the hybrid peptides against proteases hydrolysis, TIH3F was incubated with trypsin at regular intervals over a 24 h period, and analyzed using reverse phase high performance liquid chromatography (RP-HPLC). As showed in Fig. 4, after incubation with trypsin for 6 h, absorbance of TIH3F slightly dropped from 600 mAU to 500 mAU, indicating a little degradation by tryspin (Fig. 4E and F). When the incubation time prolonged to 12 h and 24 h, there were still a mass of intact TIH3F remained in the solution (Fig. 4G and H), indicative of the significantly improved resistance of TIH3F against trypsin hydrolysis compared with Hc-3, the majority of which was rapidly digested by trypsin in 6 h (Fig. 4A–D). Subsequently, the antimicrobial effect of TIH3F was investigated after *in vitro* degradation assay using three representative strains (Fig. 3E). After treatment with trypsin or proteinase K, Hc-3 almost lost its bactericidal ability, while TIH3F showed negligible antimicrobial activity decrease against *E. coli*, *S. aureus*, and *C. albicans*.

### Membrane permeabilization assay

**Figure 5:**
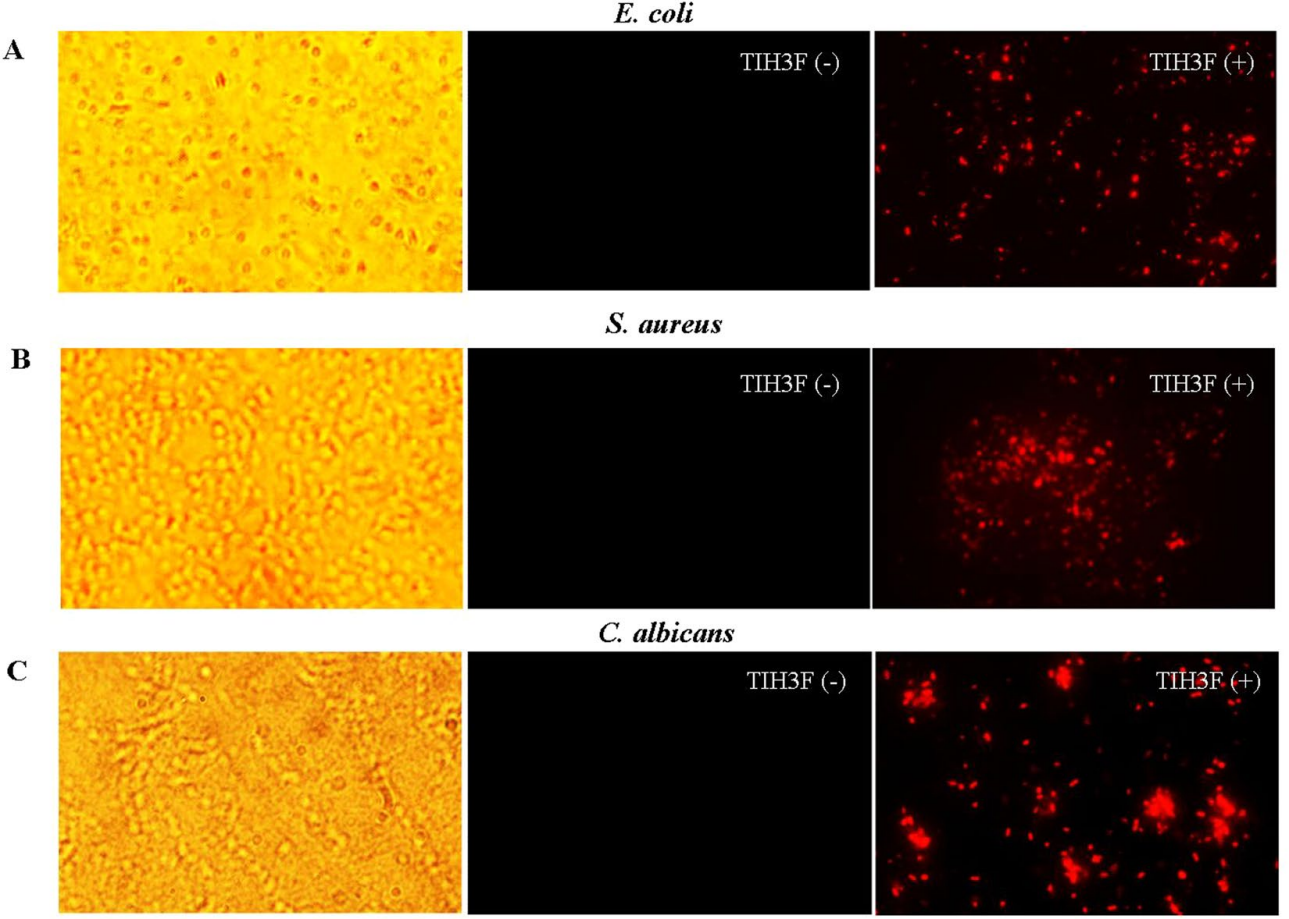
Membrane permeabilization of hybrid peptide on *E. coli* (**A**), *S. aureus* (**B**) and *C. albicans* (**C**). The microorganisms were treated with presence (+) or absence (−) of TIH3F and PI staining.

Our previous work has demonstrated that Hc-CATH mainly assumes an amphipathic α-helical conformation^31^ important for the cationic and amphiphathic AMPs to exert antimicrobial activity via permeabilization of the cytoplasmic membrane. The permeabilization causes the disruption of microbial cell integrity, leading to an obvious cell morphological alteration^37^. This mechanism makes it difficult for microorganism to develop drug resistance^2^. Here, to evaluate whether the structural modifications in current study may influence the antimicrobial mechanism of hybrid peptides, the membrane permeabilization effect of TIH3F upon *E. coli, S. aureus* and *C. albicans* were tested using the classical DNA-binding dye PI. As shown in Fig. 5, the red color from red dye that stains only the dead cells with broken membranes was significantly increased, manifesting that the membranes of microorganisms have been disrupted by TIH3F in a mode similar to that of other amphipathic alpha-helical AMPs.

### Anti-inflammatory activities of the hybrid peptides

**Figure 6:**
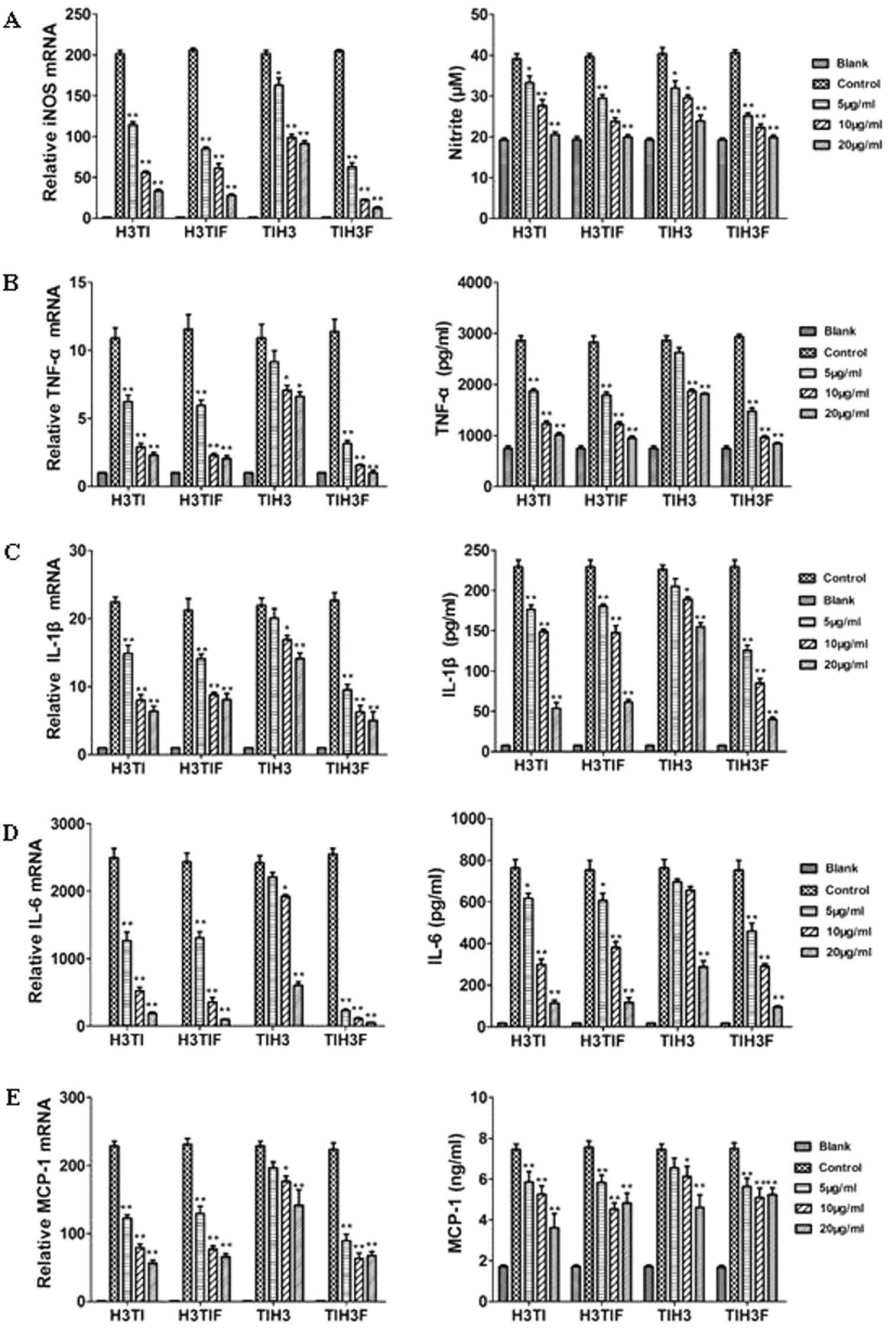
Effects of four hybrid peptides on the inflammatory factors: (**A**) iNOS & nitrite; (**B**) TNF-α; (**C**) IL-1β; (**D**) IL-6; (**E**) **MCP-1** transcription and production induced by LPS. Expression of the target genes were measured by q-PCR and normalized against the expression of GAPDH. Target genes expression in untreated cells was normalized to 1. The secretions of NO, cytokines and chemokine in RAW 264.7 culture supernatant were determined by Griess reagent and ELISA, respectively. LPS: 100 ng/mL. Data are mean ± SEM value of three independent experiments (*P < 0.05; **P < 0.01; by unpaired t test).

Local inflammation plays an important role in the early response to infection, and during this pathological process, abundant immune effectors, including NO, complement, cytokines, chemokines are recruited and participate in the recognition and destruction of invading pathogens^38^. However, among them, there are also pro-inflammatory effectors created primarily by immune cells that are engaged in the process of amplifying inflammatory reactions. Recently, emerging evidence suggests that certain members of cathelicidins have dual activities of antimicrobial and anti-inflammatory^39^. To further determine whether the hybrid peptides also can inhibit the LPS-induced inflammatory responses in addition to direct antimicrobial effect, the cytokine transcription was evaluated in macrophage cells after co-incubation with LPS and series concentrations of hybrid peptides using qPCR. The mRNA levels of the iNOS (inducible nitric oxide synthase), some key pro-inflammatory cytokines (including TNF-α, IL-1β and IL-6), and chemokine MCP-1 were all significantly upregulated after stimulation with LPS (Fig. 6). However, such increased expressions of all pro-inflammatory effectors were notably suppressed by the hybrid peptides in a dose-dependent manner. Among the four peptides, TIH3F obviously had the strongest inhibitory effect toward all pro-inflammatory effectors examined, consistent with the MIC result.

In addition, ELISA studies were also used to examine the protein level changes of cytokine and chemokine in supernatants of hybrid peptide–treated macrophage cells. All the productions of pro-inflammatory effectors greatly increased in response to LPS treatment (Fig. 6), which could be remarkably and dose-dependently suppressed by all the hybrid peptides. Coherent with the qPCR results, TIH3F had the strongest anti-inflammation effect among the four peptides. It is acknowledged that the excessive and prolonged inflammation comes at a potential detriment, as sepsis can be triggered causing massive damage and even death^40^. Therefore, the results demonstrated that the current four hybrid peptides not only could effectively kill moderate loads of incoming pathogens, but also suppress the inflammation-induced tissue injury.

## Disscusion

Over the last two decades, extensive use of traditional antibiotics has fostered the emergence and expansion of drug-resistant microorganisms, which seriously threaten the public health. Therefore, the discovery of novel classes of antimicrobial agents to fight infectious diseases is greatly needed. Cathelicidins have become the research focus of novel antimicrobial agent candidates for its broad spectrum in killing microorganism, and a variety of other functions, such as chemoattraction of immune cells, neutralization of LPS, wound healing promotion, and induction of angiogenesis^41, 42^. Besides, the unique antimicrobial mechanism of overwhelming and irreversible disruption of cell membranes further enables cathelicidins to be promising antimicrobial agent candidates, in that the special mode makes it difficult for microorganism to evolve resistance^43^. In spite of all these attractive characteristics, ongoing negative opinions limit more widespread acceptance of peptide-based drugs including cathelicidins. Therefore, a major future challenge will be the modification of peptide to solve these hurdles before the ultra clinical application, such as incensement of antimicrobial activity, enhancement of the conditional stability and enzyme tolerance, and reduction of hemolytic activity and the undesirable cytotoxicity^44–46^. Among them, the proteolytic instability is one of the biggest disadvantages of peptide drugs. So far, only a few chemical means of the amide bond and the side-chains alterations are demonstrated to render the derivatives resistant to proteolytic degradation^47^. However, there are many difficulties in the rational design of optimized peptide having the excellent performance regarding all these aspects, in that the above mentioned sequence modification have not been strictly correlated to antimicrobial activity. Therefore, an appropriate balance among all parameters may be required for the design of an ideal AMP with an increased therapeutic index.

Truncation is a common strategy to obtain the core element of cathelicidins, with shorter sequence while maintaining the desired antimicrobial activity. Combination is a simple and effective way to design novel AMPs because hybrid peptides may inherent the advantages of parental peptides^48^. It is acknowledged that the cationic, amphipathic helixes are possibly required for most AMPs to exert the antimicrobial activity via inducing the membrane permeability, although the underlying precise mechanism still remains unclear^49, 50^. Therefore, the α-helix section of Hc-CATH, our previously identified cathelicidin from sea snake was firstly truncated (Hc-1), on the basis of which a series shortened variants Hc2~Hc15 were further developed. The exploration of their antimicrobial activities demonstrated that certain length of α-helix is required for their bactericidal performances (Table 1). Thus, the length of the peptides should also be taken into consideration when designing AMPs, although this property is not as important as other physicochemical, and structural characteristics of peptides. Among Hc1~Hc15, the most prominent analog, Hc3, was selected as one of the parent templates for developing hybrid peptides. Although it is reported that the incorporation of positively charged amino acids, especially at terminal positions could improve cell and tissue penetration of peptides^51^. The current structure and activity study suggested that Phe is more important than cationic Arg and Lys at the peptide terminal in increasing its bactericidal activity. Thus, after getting two hybrid peptides (H3TI & TIH3) by combining the Hc-3 with trypsin inhibitor, ORB-C, the point mutation of strong hydrophobic amino acids such as Leu and Val with Phe was exploited to obtain the other two hybrid peptides (H3TIF & TIH3F).

Most identified cathelicidins are reported to adopt a random coil structure in aqueous solutions and assemble into a more rigid α-helix structure in a membrane-mimetic environment. The secondary structures of the hybrid peptides were determined in both solutions, and they exhibited the same characteristic of α-helical conformation as Hc3 (Fig. 1). Meanwhile, the three dimensional structure of hybrid peptides predicted by *De novo* modeling also revealed the same structure patterns. Although the results indicated that the four hybrid peptides designed were inherently cationic, amphiphilic and helical-structured, they showed compromised antimicrobial activities compared with their parental sequence of Hc3 due to the additional loop structure (Tables 1 and 4).

Different hybridizing modes of Hc3 and ORB-C seem to be important to the antimicrobial activity but not to the trypsin inhibition activity of hybrid peptide, judging by the observation that H3TI has a much broader antimicrobial spectrum than TIH3. In this case, the terminal cationic Lys in H3TI facilitates the interaction of peptide with bacterial membrane more easily than TIH3. However, after the point mutation with Phe, TIH3F that has the trypsin inhibitor loop at N-terminal shows a broader spectrum. It might be assumed that Phe residue contributes more and may even reverse the effect of terminal cationic residue in peptide’s killing activity. Nevertheless, the trypsin inhibitor activity of four hybrid peptides was not influenced by the hybridizing mode, but only related with the intact ORB-C loop independent of the other segment of peptide. Subsequently, the bactericidal kinetics data showed that all four hybrid peptides exerted killing effect faster than Hc-CATH^31^, which is probably owing to their shorter sequence that could easily and rapidly access and insert into the bacterial lipid bilayer^52^. Such rapid bactericidal action provides a remedy for their development as a novel bactericidal biomaterial.

One main barrier for the clinical administration of peptide and protein-based drugs is the enzymatic barrier, which is mediated by innate or environmental proteolytic enzymes. The main purpose of current study is to design novel cathelicidin-derived AMPs with improved protease stability. Herein, we conjugate the best truncated form of Hc-CATH, Hc3, covalently with the smallest trypsin inhibitor, ORB-C. *In vitro* enzyme assays clearly demonstrated the strong trypsin inhibition capability of the novel hybrid peptide, with the inhibition constants (*Ki*) determined to be ranging from 1.5 to 5.5 μM.

Another big problem commonly associated with clinical applications of cathelicidins is their hemolysation and cytotoxicity of mammalian cells. Therefore, in current study, the hemolytic and cytotoxic effects of the designed hybrid peptides were evaluated. At a concentration up to 200 μg/mL, the four hybrid peptides each only induced 3~10% hemolysis (Table 2) and showed neglectable cytotoxicities (data now shown), which are comparable with Hc-CATH, indicating that the extra trypsin inhibitor loop does not affect their selectivity towards the anionic microbial cell membranes over zwitterionic mammalian cell membranes, possibly due to the maintained amphiphilicity of these peptides^46^.

It is reported that the presence of salt and serum may influence the antibacterial activity of AMPs^53^, thereby the salt and serum stability assays were usually considered the most important secondary screening in peptide drug development^54^. In fact, serum stability can provide a strong prediction of the all important pharmacokinetic behavior of drugs^55^. In this study, TIH3F, the one with the best killing effect among the four hybrid peptides showed similar light susceptibility to the addition of salt with the parental Hc-CATH^31^ (Fig. 3A), and killing effect of TIH3F merely decreased by half as the NaCl concentration raised from 100 mM to 400 mM, indicating its good salt-resistance. It is acknowledged that attraction and attachment of cationic portions of the peptide to the negatively-charged microbial surfaces is regarded as the first step for AMP-mediated cell killing, so this decrease is possibly ascribed to the disruption of electrostatic attraction resulting from membrane binding competition between the peptides and cations^56^. Besides, TIH3F displayed far better serum stability in serum than Hc-CATH, and kept 65% antimicrobial activity after 36 h serum incubation, whilst Hc-3 was almost proteolyzed and lost its bacterial inhibitory activity (Fig. 3B). Indeed, such incredible serum stability of TIH3F is attributed to its trypsin inhibitor loop, the disulfide bridge of which is reported to be more resistant to protease degradation than its linear counterpart^57^. Besides, as seen from Fig. 3E and F, the incorporation of ORB-C significantly improved tolerability of TIH3F challenged by trypsin and proteinase K, suggesting the great potential of current hybrid peptide for systemic therapeutic applications. Moreover, TIH3F was highly stable within either wide pH range from 1.0 to 11.0, or in a very long time up to 96 hrs under body temperature of 37 °C (Fig. 3C and D). In addition, the degradation of the peptides was continuously monitored by RP-HPLC over a series of time spans. TIH3F showed very little sign of degradation during the first 6 hours, and still remained half up to 24 hours (Fig. 4). All of these results imply that TIH3F has a potential to develop into topically used anti-infective agent or biomaterial, which can be administered on skin and mucosa, including oral, respiratory and genital tract mucosa. However, since all experiments were carried out *in vitro*, the evaluation of hybrid peptide for clinical use requires further *in vivo* investigations.

In view of the 2D and 3D structures, all the hybrid peptides displayed a typical α-helical conformation in the membrane-mimicking environment (Fig. 1), indicative of the possibility that these peptides might exert antimicrobial activity via bacterial membrane permeabilization. To test this hypothesis, the membrane permeabilization effect of TIH3F upon representative G+, G− bacteria and fungi were performed. As expected, TIH3F of 5 × MIC induced much more fluorescence leakage than untreated group (Fig. 5), suggesting that TIH3F caused morphological damage of the microorganism membranes, followed by the cytosolic leakage and whole cell lysis. This also can explain why cathelicidins are effective to those drug-resistant strains. The anti-biofilm assay further confirmed that TIH3F could destroy the *C. albicans* biofilm, and even effectively kill *C. albicans* in preformed biofilms (Fig. 2), via a mechanism associated with pore-formation and membrane-lysis^58^.

Inflammation is a mechanism for maintaining homoeostasis in response to detrimental stimuli like infection. Limiting the inflammation-induced tissue injury can simultaneously enhance the AMPs’ protective ability^59^. In current work, the four hybrid peptides were determined to inhibit the LPS-stimulated inflammatory cytokines (TNF-α, IL-1β and IL-6) and chemokine (MCP-1) production both on mRNA and protein level, with TIH3F exerting the best inhibitory effect, consistent with the MIC result. These effects might result from the neutralization of LPS by cathelicidin, which binds and neutralizes the anionic amphiphilic lipid A domains of LPS via ionic interactions, and dissociates LPS aggregates via hydrophobic interactions between the LPS’s alkyl chains and non-polar amino acid side chains^17^. Considering that hybrid peptides have a high positive charge of +10 and hydrophobicity (Table 3, Fig. 1B), it is rational for them to show the strong LPS neutralization activities. In addition, it is stated that cathelicidin also binds to the opening region of the MD-2 pocket of TLR4-MD-2 complex, and subsequently blocks LPS entering into the pocket, resulting in the inhibition of the TLR4 signaling pathway activation^39^.

In conclusion, four novel peptides were designed and optimized by means of hybridization and point-mutation, in order to enhance the biochemical stabilities, and most importantly, to conquer the sensitivity to proteolytic degradation, while increase or at least maintain their antimicrobial potency and cellular selectivity. Of the four designed peptides, TIH3F exhibited the best antimicrobial and anti-inflammatory activities, and much enhanced stabilities in the presence of broad spectrum proteases, making it a good candidate as a safe, stable and effective biomedical agent for future clinical applications. Moreover, this work also indicates that the construction of hybrid peptides is an attractive approach for the optimization of protein/peptide-based molecules.

## Methods

### Design and optimization of the hybrid bifunctional peptides

In our previous work, a novel cathelicidin (Hc-CATH) was characterized from the sea snake *Hydrophis cyanocinctus*. Hc-CATH is composed of 30 amino acids, and its sequence is KFFKRLLKSVRRAVKKFRKKPRLIGLSTLL^31^. Structural analysis indicated that Hc-CATH mainly adopts an amphipathic α-helical conformation in bacterial membrane-mimetic solutions. It possesses not only potent and broad-spectrum antimicrobial activity but also strong anti-inflammatory activity by inhibiting the LPS-induced production of nitric oxide (NO) and pro-inflammatory cytokines^31^. Thus, in current study, Hc-CATH was exploited as one of the original templates for designing the hybrid peptides, especially considering its stability and ignorant cytotoxicity.

Afterwards, the α-helix segment of Hc-CATH was truncated, on the basis of which a series shortened variants of 1 residue shorter were developed from C and N terminal, termed Hc1~7 and Hc8~13, respectively, whilst Hc14 and Hc15 were designed by cutting off residues from both terminals of the α-helix. The 15 peptides were chemically synthesized and characterized by antimicrobial activity against various bacterial species and hemolytic activity against hRBCs. Afterwards, the one that exhibited highest cell selectivity towards bacterial cells over hRBCs was chosen to be one parental peptide for designing the hybrid bifunctional peptides, which were subsequently engineered with the ORB-C, a disulfide bridged hendecapeptide proteinase inhibitor loop derived from skin of frog (*Odorrana grahami*), at its N- and C- terminals, respectively.

Next, based on the activity screening of the Hc1~15, the structure-activity relationship was analyzed in order to identify the key amino acid residues important for their antimicrobial activity. Then the hybrid peptides were further modified by replacing certain residues with the key residue before final chemical synthesis. Physicochemical properties of hybrid peptides were calculated using ProtParam tool (http://web.expasy.org/protparam/) and the secondary structures were predicted online by The PSIPRED Protein Sequence Analysis Workbench (http://bioinf.cs.ucl.ac.uk/psipred/). Peptides’ amphipathy was demonstrated by helical wheel projection (http://heliquest.ipmc.cnrs.fr/cgi-bin/ComputParamsV2.py#userconsent#)

### Peptide synthesis

All peptides designed were synthesized using a peptide synthesizer from GL Biochem (Shanghai) Ltd. (Shanghai, China) and analyzed by HPLC and MALDI-TOF MS to confirm that the purity was >95%. All peptides were dissolved in double distilled water and used for following experiments.

### Structure modeling

Circular dichroism (CD) spectroscopy was performed to evaluate the secondary structure of the designed hybrid peptides in solvent environments. CD spectra were recorded at 298 K on a Jasco J-715 spectrophotometer (Jasco, Japan). Samples were prepared by dissolving peptide to an ultimate concentration of 0.2 mg/mL in two different solvents, water and 50% (v/v) trifluoroethanol(TFE)/water, respectively, and then added into a quartz optical cell at 25 °C. The spectra of wavelengths ranging from 190–260 nm was measured, the instrument parameters were: 0.1 cm path-length cell, 1 nm bandwidth, 1 sec response time, a scan speed of 100 nm/min. The spectra were averaged over three consecutive scans, followed by subtraction of the CD signal of the solvent.

The three dimensional structure of the hybrid peptides were modeled by homology. A BLAST search with default setting was performed in the Protein Data Bank (PDB) to obtain the suitable templates. Multi-templates were selected for each peptide in order to generate the model as accurate as possible. Based on the similarities and coverage with H3TI and TIH3, the crystal structures of PDB entry 4EWC (61% identity, query cover 50%) by X-ray diffraction was exploited as a template for homology modeling. Similarly, the crystal structures of PDB entry 2X07 (67% identity, query cover 42%) and 1DCH (69% identity, query cover 42%) by X-ray diffraction were used as templates for H3TIF and TIH3F, respectively. The tertiary structures of four peptides were generated and optimized using MODELLER (version 9.10). All of the three dimensional models were visualized by Pymol software (version 1.7.6) without any other refinements.

### Antimicrobial assay

A two-fold broth microdilution method described in our previous paper was used to determine the antimicrobial activities of parental and hybrid peptides^39^. More than 30 strains including Gram-negative bacteria, Gram-positive bacteria and fungi were used in the current assay, in which the standard strains were stored in our lab and the clinical strains were collected from local hospitals. Briefly, bacteria were incubated in Mueller-Hinton broth (MH broth) at 37 °C to exponential phase and diluted to 10^6 ^CFU/mL. A serial dilution of peptide sample were prepared in 96-well microtiter plates (50 μL) and mixed with equal volume of microbial inoculums (50 μL). The plate was slowly shaken in the microbial incubator, and measured at A595 after incubation at 37 °C for 18 h. The minimal concentrations at which no microbial growth occurred were recorded as MIC (minimal inhibitory concentration) values. The traditional antibiotic ampicillin was used as positive control. The results are shown in an average of the MICs obtained from three independent experiments.

### Bacterial killing kinetic assay

The bacterial killing kinetics of the hybrid peptides against *E. coli* 08040726 was determined by counting the changes in the viable bacterial clones after peptide treatment. The experiment was carried out according to the method described previously with minor modifications^36^. Fresh colonies of the bacteria were incubated in MH broth at 37 °C for 12 h and diluted to 10^6^ CFU/in fresh MH broth. The hybrid peptide was added to the bacterial suspension with a final concentration of 1x and 5x corresponding MICs, and incubated at 37 °C for 0, 10, 20, 30, 45, 60, 90, 120, and 180 mins. At each time point, aliquots (50 μL) were removed and diluted with fresh MH broth for 1000 times. 50 mL of the dilutions were coated on MH agar plates, incubated overnight at 37 °C, and the viable colonies were counted. Ampicillin was used as positive control and sterile deionized water was used as negative control.

### Effect of hybrid peptide on *C. albicans* biofilms

TIH3F that has the best antifungal activity among the four peptides was chosen to examine the effect on the *C. albicans* biofilm formation and the established biofilm using previously described method with minor modifications^60^. In the biofilm inhibition assay, after the incubation at 35 °C, *C. Albicans* was diluted to 1 × 10^6^ CFU/mL in RPMI-1640 medium. Then 2 mL culture was added to each well of 6-well plates with coverslips at the bottom, and incubated at 35 °C in a humid chamber for 3 h. Non-adherent cells were then washed away by PBS, and series concentrations of TIH3F (0, 1, 2, 4 μg/mL) were added into the plate, with amphotericin B as control. The formation of the biofilm was determined by the XTT Cell Proliferation and Cytotoxicity Kit (KeyGEN BioTECH, Nanjing, China) after 36-h incubation. In the biofilm disruption assay, the biofilm was preformed as described above but with a longer incubation time up to 72 h. Then TIH3F and amphotericin B of series concentrations (0, 2.5, 5, 10, 25, 50 μg/mL) was added after non-adherent cells were washed out. The measurement of remaining biofilm was then determined. In order to further confirm the antifungal effect of TIH3F against *C. albicans* encased in biofilms, the established biofilm was treated with TIH3F (0, 5, 50 μg/mL), washed three times with PBS, and finally stained with 1 mL 1000-fold diluted PI/SYBR Green I at 30 °C in the dark for 20 min. After washing with PBS, the plate was immediately photographed with fluorescence microscope.

### Hemolytic and cytotoxic assays

In order to determine hemolytic activity of the peptides, the amount of hemoglobin released from lytic human erythrocytes was measured by monitoring the absorbance at 540 nm, using 1% Triton X-100 (v/v) as a positive control^10^. *In vitro* cytotoxicities against RAW264.7 murine macrophage cells and HUVECs were determined by MTT described previously^39^. All experiments were repeated three times.

### Proteinase inhibition assay

The inhibition effect on the hydrolysis of synthetic chromogenic substrate by trypsin was assayed in 50 mM Tris–HCl, pH 7.8 buffer at room temperature. The trypsin of final concentration of 0.1 mg/mL and different amounts of the hybrid peptide (final concentrations ranging from 0 to 0.16 μg/mL) were pre-incubated for 5 min at 37 °C. N-α-Benzoyl-DL-arginine p-nitroanilide hydrochloride (NBLAN) was used as a substrate for trypsin. The reaction was initiated by the addition of NBLAN with a final concentration of 48, 96 and 192 μg/mL, respectively. The formation of yellow product, p-nitroaniline was monitored continuously at 405 nm^61^. The inhibition constant *Ki* of the hybrid peptides were determined according to the method of Dixon (1953)^62^.

### Stability of hybrid peptides

#### Salt stability

To determine the effect of salt on the antimicrobial activity of the parental and hybrid peptides, the bacteria colonies survived 1 × MIC of peptide in the presence of salt were counted. Briefly, *E. coli* ATCC25922 were diluted into decent concentration and mixed with each kind of peptide solution of 1 × MIC supplemented with NaCl (0, 50, 100, 150, 200 and 400 mM), respectively. Aliquots were removed, diluted and plated on nutrient agar plates. The number of colonies after incubation at 37 °C for 18 h was counted. The salt stability of the peptides was assessed by the number of colonies.

#### Serem stability

Serum stability of Hc-3 and hybrid peptides was examined as follows: 0.8 mL of peptide sample (2 mg/mL) was incubated with 0.2 mL of freshly prepared human serum at 37 °C for 0, 3, 6, 12, 24 and 36 h, respectively, and then the bacteria colonies survived 1 × MIC of peptide were recorded at each time interval.

#### Protease stability

To further examine the stability of TIH3F incubated with enzymes (trypsin and protease K) over time, the LC analysis of peptide degradation over a series of time spans (0, 6, 12, 24 h) was performed. TIH3F and protease solution were prepared in 0.1 M NH_4_HCO_3_ buffer (pH 8.2) to final molar ratio of 300:1, and incubated at 37 °C for 6, 12, and 24 h, respectively. Then 150 μL aliquots were taken at given time intervals, diluted with 150 μL of water/acetonitrile (60/40 v/v) containing 2% TFA, and analyzed by RP-HPLC (Thermo Scientific Syncronis C_18_ column, 250 mm × 4.6 mm, 5 μm), eluted with acetonitrile/water (containing 0.1% TFA) mixture of linear gradient concentration^24^.

On the other hand, the antimicrobial activity of peptides after proteases incubation were determined as follows: Hc-3 and hybrid peptide were individually incubated with trypsin or proteinase K (mol/mol: 300/1) for 30 min at 37 °C^63^, and then mixed with bacterial (*E. coli*, *S. aureus* and *C. albicans*) at final concentration of 1 × MIC for 2-h more incubation. Aliquots were removed, diluted and plated on nutrient agar plates. The numbers of colonies after incubation at 37 °C for 18 h were counted. The system with proteinase alone served as blank, and Hc-3 was used as negative control.

#### pH stability

Peptide was dissolved in HCl solution or NaOH solution to a final concentration of 2 mg/mL with pH value ranging from 1.0 to 11.0, and incubated at 37 °C for 24 h^64^. The peptide were added to bacterium suspension until final concentration of 1 × MIC and incubated for 2 h at 37 °C. The diluted aliquots were seeded at MH agar plates and the CFU were scored after 24 h incubation at 37 °C. The number of CFU grown out of MH broth without peptide was served as control.

#### Thermal stability

Thermal stability of Hc-3 and hybrid peptides was examined according to the method described in our previous work^31^. Peptide was dissolved in sterile deionized water to a final concentration of 2 mg/mL, and incubated at 37 °C for 0–96 h. At each time point, the bacteria colonies of *E. coli* ATCC25922 survived 1 × MIC were measured.

### Membrane permeabilization assay

Propidium iodide (PI), a kind of DNA-binding fluorescent dye, was used to examine the membrane permeabilization effect of hybrid peptides^39^. Briefly, *E.coli* ATCC25922 was cultured to exponential growth phase in MH medium, collected by centrifugation, and then diluted to OD_600_ of 0.5 with PBS. The peptide solution was added to bacterial suspension at a final concentration of 5 × MIC with the presence of PI of 10 μg/mL. After 30 min incubation at 25 °C, the cells were washed 3 times with PBS and immediately photographed using fluorescence microscope (Olympus, Japan).

### Determination of NO and pro-inflammatory cytokines

Murine macrophage Raw264.7 cells (3 × 10^5^/well) were treated with series concentrations of hybrid peptides and LPS (100 ng/mL) for 6 h. Total RNA was isolated using RNAiso Plus kit (Takara) and synthesized into cDNA with PrimeScript Reverse Transcriptase (Takara). The cDNA was assessed by q-PCR using SYBR green master mix kit (Takara) on a Realplex Mastercycler (Eppendorf, Germany). Gene expression was calculated after being normalized to those of GAPDH levels by Δ Δ Ct method27. The accuracy of the amplification reactions was achieved by melt curve analysis.

RAW264.7 cells were cultured in DMEM (Gibco, Gaithersburg, USA), containing 10% fetal bovine serum, 100 U/mL streptomycin and 100 U/mL penicillin at 37 °C with 5% CO_2_, and plated in 24-well plate (1 × 10^6^ cells/well). After adhesion, the cells were treated with LPS (100 ng/mL, from *E. coli* 055:B5, Sigma-Aldrich) in the absence or presence (5, 10, 20 μg/mL) of peptides for 24 h in the same condition. Untreated cells and LPS alone treated cells served as control. The NO was determined using Griess reagent (Beyotime, Jiangsu, China) according to the manufacturer’s protocol. ELISA kits (Joyee, shanghai, china) were used to detect the levels of pro-**i**nflammatory cytokines and chemokines in RAW264.7 cell culture supernatant, including TNF-α, IL-6, IL-1β and MCP-1.
